# Screen Recordings as a Tool to Document Computer Assisted Data Collection Procedures

**DOI:** 10.5334/pb.490

**Published:** 2019-07-16

**Authors:** Tobias Heycke, Lisa Spitzer

**Affiliations:** 1 GESIS – Leibniz-Institute for the Social Sciences, DE; 2 University of Cologne, DE

**Keywords:** replication, screen recording, documentation, reproducibility

## Abstract

Recently in psychological science and many related fields, a surprisingly large amount of experiments could not be replicated by independent researchers. A non-replication could indicate that a previous finding might have been a false positive statistical result and the effect does not exist. However, it could also mean that a specific detail of the experimental procedure is essential for the effect to emerge, which might not have been included in the replication attempt. Therefore any replication attempt that does not replicate the original effect is most informative when the original procedure is closely adhered to. One proposed solution to facilitate the empirical reproducibility of the experimental procedures in psychology is to upload the experimental script and materials to a public repository. However, we believe that merely providing the materials of an experimental procedure is not sufficient, as many software solutions are not freely available, software solutions might change, and it is time consuming to set up the procedure. We argue that there is a simple solution to these problems when an experiment is conducted using computers: recording an example procedure with a screen capture software and providing the video in an online repository. We therefore provide a brief tutorial on screen recordings using an open source screen recording software. With this information, individual researchers should be able to record their experimental procedures and we hope to facilitate the use of screen recordings in computer assisted data collection procedures.

## Introduction

*[…] we thereby admit that no isolated experiment, however significant in itself, can suffice for the experimental demonstration of any natural phenomenon […]* – Fisher (1974), p. 13.

As the quote by Fisher demonstrates, in the empirical sciences, we need to replicate findings (i.e., repeat a study) in order to gain certainty about the existence of natural phenomena. In recent years, many scientific fields have re-discovered the need for replication of scientific studies. Replication studies are discussed in sport science (Halperin, Vigotsky, Foster, & Pyne, 2018), economics (Camerer et al., 2016), and marketing (Hunter, 2001). Famously, in a large replication project in psychology only approximately 40 % of the selected 100 studies were replicated by independent researchers (Open Science Collaboration, 2015). Additional (large-scale) replication attempts in psychology have confirmed initial findings that many published findings could not be replicated independently (e.g., Hagger et al., 2016; R. Klein et al., 2014). These results have led some to speak of a “replication crisis” in the psychological sciences (Schooler, 2014). The term “replication crisis” even has its own Wikipedia page (https://en.wikipedia.org/wiki/Replication_crisis) and has been covered by popular media outlets (e.g., Resnick, 2018; Yong, 2018).

Generally, one could propose two main reasons why a published finding cannot be replicated: First, the initial finding was merely a coincidence and the reported effect was not describing a natural phenomenon. Possible reasons for such a finding could be false positive statistical findings (which were potentially increased by *p*-hacking or other means of data massaging, Simmons, Nelson, & Simonsohn, 2011). Second, a finding might be based on a genuine phenomenon, but the effect was not replicated because essential details from the original experimental^1^ procedure were altered (assuming that the replication attempt had sufficient statistical power). In the following, we will discuss this argument and propose an easy solution to document one’s experimental procedure in order to empower replication studies to mimic the original procedure as closely as possible.

## Experimental procedure documentation

When reproducing a study procedure as closely as possible in a replication attempt, we often speak of a direct replication attempt. While direct replications are practically not possible as time and participants change (S. Schmidt, 2009), we will use the term ‘direct replications’ to describe ‘as close as possible’ replication attempts, where the researcher “should strive to avoid making any kind of change or alteration” (Earp & Trafimow, 2015, p. 5). Of course changes in the procedure might be unavoidable when measuring or manipulating a concept that changed since the data collection of the original study (Brandt et al., 2014). Direct replications might be highly informative, especially when the original results cannot be replicated in independent replication attempts. Specifically, not finding the original effect with a different method can easily be attributed to the method rather than the original effect (Doyen, Klein, Simons, & Cleeremans, 2014). Therefore, when a replication attempt does not succeed to find the original pattern of results, researchers might speculate whether the difference in the results could be due to *subtle differences in the experimental procedure* or *critical changes* that were introduced by the replicators (Freese & Peterson, 2017).

In (psychological) science post-hoc arguments can always be given to argue why studies could not replicate original findings. One might therefore be tempted to dismiss any post-hoc argument explaining why a replication attempt might have failed. However, what if effects indeed depend on small changes to the experimental procedure that are -so far- not understood by the scientific community? There are some reports of such instances: In a replication attempt of the *verbal overshadowing* effect (Alogna et al., 2014), a small adjustment to the experimental procedure (i.e., length and order of the filler task) was erroneously introduced. The replication study deviated from the original study protocol in such a small detail, that it was not discovered by the original and replicating authors when vetting the study protocol. The replication was repeated adhering to the original procedure and indeed, the effect size found when adhering to the original procedure was similar in size to the original effect size and larger than the effect size of the replication with the small changes.

Additionally, Collins (1985) illustrates the problem of minor details in experimental settings with an example from physics: A physicist, who had built a specific laser previously, attempted to build the same laser again. Even though he had access to the plans and possessed the ‘tactical knowledge’ that is not included in the written report, he needed to make approximately 20 adjustments to his finished product before the laser was actually working. This example, while far from (experimental) psychological procedures, nicely demonstrates a problem in psychological replication research: Unlike constructing a laser, where we know that if all parts are installed correctly it will produce a light beam, in psychological science we do not know whether the effect is real or not (i.e., whether the alternative or null hypothesis is true). If we make 20 different adjustments and run the experiment after each adjustment (similar to attempting to start the laser after each adjustment), we should expect to observe at least one statistically significant effect even when there is no true effect (if we accept an alpha error of 5 % in every statistical test). We therefore need to ensure that we conduct high quality replication attempts in psychology.

To conclude, it should be considered highly problematic if small changes might lead to differences in the outcomes of an experiment, especially when researchers are potentially not aware of which details might be important and which not. If this is indeed the case, these details might therefore not be reported in the written manuscript and we can only speculate if a non-replication might depend on one of these details. It is, however, simply not feasible to repeat an experiment with all combinations of potentially important methodological details.

If methodological details are missing from the written report, it might not only be problematic in a replication attempt but also during the peer review process. Details of the experimental procedure might not be mentioned in the manuscript, that could result in a more critical assessment of the method by peer reviewers. One could imagine that a peer reviewer who is an expert on scales (e.g., for an evaluative measure) might not be able to critically comment on a manuscript that merely mentions that ‘an evaluation was measured on a 100 point scale’, as different scale layouts can result in different response patterns by participants. Additionally, even if a method is described in detail, it might be difficult for a reviewer to fully understand the procedure. A highly detailed description of the experimental procedure might even be overwhelming and therefore prevent a reader from understanding the method. For a critical and in depth peer review process, a detailed description – that is easy to understand – would be ideal.

Concluding, an author might not be aware which (small) details might be important in the experimental procedure and might not include these details in the written report (i.e., the published manuscript). These details, however, might change the outcome of the peer review process or replication attempts substantially. Based on the potential effect on the peer-review process and replication attempts, we conclude that there is a need to document one’s experimental procedure as closely as possible to facilitate replication efforts and peer review control.

## A possible solution

One recommendation that appears to solve the above-mentioned problems, is to provide the research material to reviewers and post them publicly after publication (Asendorpf et al., 2013; Lindsay, 2017). The transparency and openness promotion (TOP) guidelines for example propose that “Materials must be posted to a trusted repository, and reported analyses will be reproduced independently before publication” as the highest level of transparency of research materials (Nosek et al., 2015, p. 1424). These materials need to include the experimental script (i.e., the code of the experimental procedure) and all necessary stimulus files. In theory, uploading all materials to a public repository would solve most problems discussed in the previous section.

However, in our opinion, there are a number of potential problems related to merely uploading experimental procedure scripts and material: First, one needs to possess the software the script was written for in order to run it. Unfortunately, many software solutions that are currently used are not freely available and the scripts can therefore not be run by every independent researcher. Second, even if one owns the software, or a freeware was used, the software version might have changed and the procedure might therefore look differently or the script does not run at all. Third, even if the software is still up to date and the researchers have access to it, they might not be acquainted with it and it may be time consuming to set up the script even when detailed instructions are provided (e.g., set up folder structures, arrange operating system or additional software). Even if investing a substantial amount of time into the empirical reproduction of a data collection procedure is justifiable in a replication attempt, it is simply not feasible to invest the time as a peer reviewer. As demand for peer review is high and peer review is time consuming (Huisman & Smits, 2017; Kovanis, Porcher, Ravaud, & Trinquart, 2016), setting up the experimental script cannot be part of the peer review process in its current form. Additionally, for a lay person interested in the methods of the study, setting up the experimental software is also a very high threshold. Thus, a comprehensive way of reporting the method of a study seems necessary.

In summary, researchers are realizing how important replication efforts in (psychological) science are, but small changes in the experimental procedure might have an impact on the results of a replication study. One advocated solution, namely uploading the experimental material, might not be sufficient to give a detailed documentation of the experimental procedure. Additionally, a reviewer does not have the time to set up the experimental software to inspect the procedure. We therefore propose in the following segment that a screen recording of the experimental procedure should be provided by researchers alongside the experimental material.

## Documenting the experimental procedure with screen recordings

We propose that the experimental procedure should be recorded by means of screen capture and the video should be made available to others (e.g., by uploading it to a public repository). This way, the procedure is easy to access by reviewers, peers and lay persons interested in the procedure and researchers interested in reproducing the procedure to replicate the work. Importantly, screen recordings will not be affected by software changes, that produce a different look with the same experimental script. Additionally, researchers do not need to acquire and set up software solutions in order to have a look at the procedure that is likely more detailed than the description in the (published) manuscript. Especially referees in the peer review process would benefit highly from this documentation of the research method alongside the written report and additional material to inform themselves about the experimental procedure.

When suggesting a new method of documenting the experimental procedure, one question is naturally of high importance: How feasible is this method for the researcher documenting the procedure and how time consuming is it? As we will show below, the software can be set up easily in a matter of minutes and the time that needs to be invested by researchers recording their procedures is minimal. The benefit on the other hand is that one has a highly detailed documentation of the procedure (to the researchers themselves, as well as the outside world). As we will show below, it is additionally possible to record keyboard and mouse input, which adds more details about the actual procedure than screenshots or uploading the material, as one can see how the software reacts to input by participants. Importantly, we do not argue that screen recordings should be seen as a substitute for providing the experimental software. The experimental software and materials should, of course, still be shared publicly, along with a detailed description of the method in the manuscript, in order to facilitate replication attempts.

In the next segment we would like to provide a short tutorial on recording the experimental procedure using the open source software Open Broadcaster Software (OBS; Bailey, 2018). Of course, one can use any tool available to record the procedure but we will demonstrate it using OBS as it is an open source solution and maintained by an active community. Many other free recording tools add a watermark to the video recording, which is not the case with OBS. When using OBS for the first time, one might find the user interface rather complex as it offers a large number of possible functions and options. But since a few basic steps are sufficient for recording experiments, OBS is not as complicated to use as it seems at first sight. We will give some suggestions on the recording process, settings one could use, processing options, and uploading options (for a brief example video of how OBS works see https://osf.io/b4uyg/). Once the basic principles are clear, one might find the various possibilities within the software rather beneficial. For example, with OBS it is possible to display mouse clicks and keystroke input and sounds can be recorded as well. Additionally, one can crop certain parts of the recorded display, in order to display only the relevant section of the screen. Another advantage of OBS is that it is compatible with many commonly used experimental software solutions (we tested OBS with PsychoPy2, OpenSesame, Inquisit, MediaLab, E-Prime and web browsers). Furthermore, it can be used with a number of different operating systems (Windows, macOS and Linux). While testing, we discovered that the way OBS functions might differ slightly between these different operating systems. Thus while we give an overview on some important steps, if they do not work with your system, we encourage you to try different settings. An overview of the mentioned features is given below and a detailed, step-by-step guide is additionally available online (see osf.io/3twe9). For an overview of all relevant online sources, see Table 1. Please note that this tutorial should serve as a first starting point and should not be regarded as a definitive guide. It should be noted that we propose that the researcher (or a research assistant) completes the experimental procedure while the software is recording the screen and we do not advise to record the procedure during actual data collection.

**Table 1:** Relevant online sources, descriptions and links.

Name	Description	Link

Online Tutorial	Step-by-step guide for using OBS	https://osf.io/3twe9
Open Broadcaster Software (OBS)	Free, open source software for screen recording	https://obsproject.com/
Shotcut	Free, open source video editor	https://shotcut.org/
Using OBS – example video	Short example of how to record a window capture	https://osf.io/b4uyg/
Screen recording – example video	Example of an experimental recording uploaded to OSF	https://osf.io/eyfxs/

## Setting up OBS

Before recording, one needs to download and install the OBS software from https://obsproject.com. If you want to record keyboard and mouse input, make sure to install the plugin “browser source” during the installation process. After installing and launching OBS, the first step should be to adjust the settings. As described above, there are many possible adjustments one can make, but the most important ones to be attended concern the video output location, the recording quality, the format, and the resolution settings of the recorded videos. First, one should check the location where the videos will be saved. The recording quality should be set to high quality. Regarding the video format, we suggest to use mp4 as it is compatible with many editing software solutions and uploading platforms. Furthermore, one should adjust the base and output resolution to the monitor’s specifications and record with high fps rate (e.g., 60 frames per second) if the used system is capable of doing so, especially if timing is an important factor in the experiment one wants to record.

## Recording an experimental procedure

After adjusting the settings, one can begin preparing the experimental recording. To be able to do this, one needs to create the “sources” one wants to record. A source represents the target of the recording, for example a display (for fullscreen experiments) or window capture (for experiments running in window mode or in a browser). Imagine you want to record an experiment running in a web browser: in this case, you would click on the “add” button in the sources area of the OBS window and select to record a window capture (see Figure 1.1).

**Figure 1:**
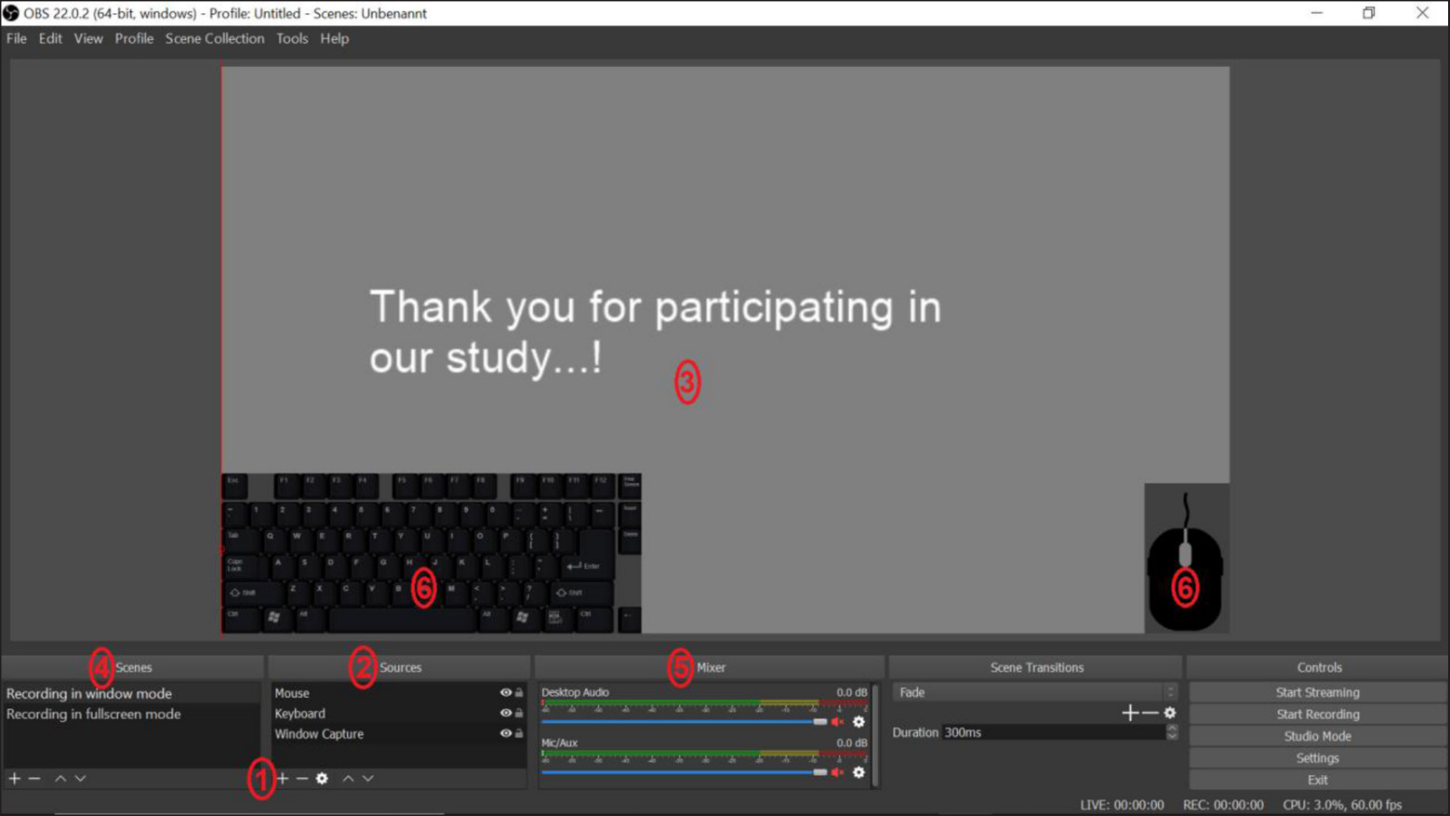
OBS window with an overview of different functions. **1)** Adding a new source by clicking on the ‘+’ button. **2)** The sources order list which can be adapted by drag and drop. **3)** The display shows everything that is currently included in the screen recording. **4)** Scenes contain different setups of sources. **5)** Audio mixer. **6)** Example of a screen recording that includes the recording of mouse clicks and keystrokes.

When creating a source, one will be asked to adjust its properties, e.g., when creating a window capture, one needs to select the target window out of a list of all windows currently open. Furthermore, it is possible to capture the cursor (mouse clicks can be recorded differently, as explained later). By right-clicking on a source, one can also adjust advanced settings for each source. We want to highlight two of these advanced settings because we found them to be of importance to ensure the correct recording of our experiments. First, if one wants to record an experiment running in fullscreen mode, one should select “Transform“ and “Fit to screen“ in the advanced settings list. This is important because it ensures the experiment to be presented correctly in the actual video – if this option is not selected, part of the video screen might remain black because the recording was not transformed to fit to the screen. Secondly, by using the “Filters” function, which can be important when recording in window mode, one can crop the recorded window. For example when recording a web browser experiment, elements such as bookmarks or the navigation bar can be removed using the crop option (a more detailed explanation of creating the filter and using the crop option can be found in the online tutorial: osf.io/3twe9).

All previous steps concerned the set-up of one single source which might be sufficient for a simple experimental recording technique. At a later stage, you might want to create multiple sources (e.g., when recording and displaying mouse clicks and keystrokes, these need to be recorded via additional sources). All sources are organized in the sources order list (see Figure 1.2). Sources on top of the list will be recorded on the foreground, sources on the bottom of the list in the background. Therefore, mouse and keyboard sources should be moved to the top of the list as they would otherwise be hidden behind the main source (e.g., the web browser recording). Furthermore, one can set sources one currently does not want to record to invisible by clicking on the eye symbol next to each source. By doing so, the source will not be included in the recording, but can easily be re-included later by making it visible again. Everything that will be included in your recording is displayed in the screen at the center of the OBS window, thus one can easily inspect if everything that should be recorded is in fact included (see Figure 1.3).

Furthermore, it might be useful not only to implement multiple sources, but to also create multiple “scenes” (see Figure 1.4). In each scene, one can create and save a specific set of sources, thus if one has multiple scenes it is easy to switch between different setups. For example, one could create one scene for recording experiments running in fullscreen mode and one for recording experiments running in window mode. All sources and adjustments (e.g., filters) one has set up within one scene will be saved and can be easily accessed when selecting this specific scene. In addition to recording the visual footage of your procedure, it is also possible to include audio or record sound by microphone. This can be done via the audio mixer also included in the general OBS window (see Figure 1.5).

Once all sources that should be recorded are created and the settings are adjusted as described above, one can start the recording by clicking on “Start Recording“. Now one can switch to the experiment and run it. After completing the experiment, switch back to OBS and click on „Stop Recording“ – the video will be saved automatically in the folder specified in the output settings. With that, you have successfully recorded your experiment that can now be edited and uploaded, which we will describe below.

## Recording of keyboard and mouse

As mentioned above, an important advantage of sharing screen recordings of experiments is the ability to display a participant’s interaction with the software by recording mouse clicks and keystrokes. For this, an OBS add-on is required, namely DisplayKeystroke (xxdocobxx, 2018). After downloading and extracting the folder, you will find different possible layouts you can record (e.g., keyboard, number pad and mouse) in the layout subfolder. To implement these layouts in OBS, one needs to add a new source of the type “Browser” in an analogous manner as described above. When creating the browser source, one needs to select an URL for the source to access. Per default, an online URL will be suggested, but to use DisplayKeystroke, one needs to select “local file” to access a local URL. The html file that corresponds to the layout one wants to record should then be selected from the Keystroke folder. For example, if you want to record the keyboard, you would select the html file “QWERTY+mouse” (the mouse has to be added separately, even though the name suggests otherwise). The keyboard layout can be adjusted manually if QWERTY is not the keyboard layout used (for a guide see the online tutorial: osf.io/3twe9).

After creating the browser source, the keyboard will be shown in the recording screen at the center of the OBS window. In an analogous manner, one can create a source to capture the mouse and place both layouts to one’s liking by moving them within the recording screen. It might, for example, be beneficial to move both keyboard and mouse layout to the corners of the recording screen and reducing them in size to avoid covering up important elements of the experiment (see Figure 1.6). Furthermore, one should remember to move the keyboard and mouse sources to the top of the sources list (see Figure 1.2). This will ensure that they are recorded in the foreground of the video and are not covered by other footage (e.g., the web browser one is recording). Additionally, before the actual recording, one needs to open and start the “KeystrokeServer” that can also be found in the downloaded Keystroke folder. Only while this server is running, the keystrokes and mouse clicks will be recorded and shown in the video. After creating the browser sources and starting the KeystrokeServer, OBS will automatically capture the keystrokes and mouse clicks while recording the experiment (see also the OBS example video: https://osf.io/b4uyg/).

## Post-processing of the screen recording

After recording and taking a look at the recorded video, one might notice that inconveniently, not only the relevant content is included (i.e., the experimental procedure) but instead, irrelevant footage (i.e., the starting and stopping of the recording in OBS) is visible, that one should remove before sharing the video. As the recording itself, the editing can be done with a variety of different programs. We have opted for the software Shotcut (https://shotcut.org; Meltytech, LLC, 2018), as Shotcut is a free, open source software available for Windows, macOS, and Linux. Like OBS, it offers a broad range of possible functions, but a limited number of more basic functions are sufficient for editing the recording. After downloading and installing Shotcut, one can open the video file (e.g., the video of the just recorded experimental procedure). The video will then be displayed in the middle of the Shotcut window. To edit the recording, one needs to transfer it from the display screen to the area at the bottom of the Shotcut window (for this, simply drag-and-drop the video to the area below). Now, the video will be displayed in a timeline that can be edited (see Figure 2.1).

**Figure 2:**
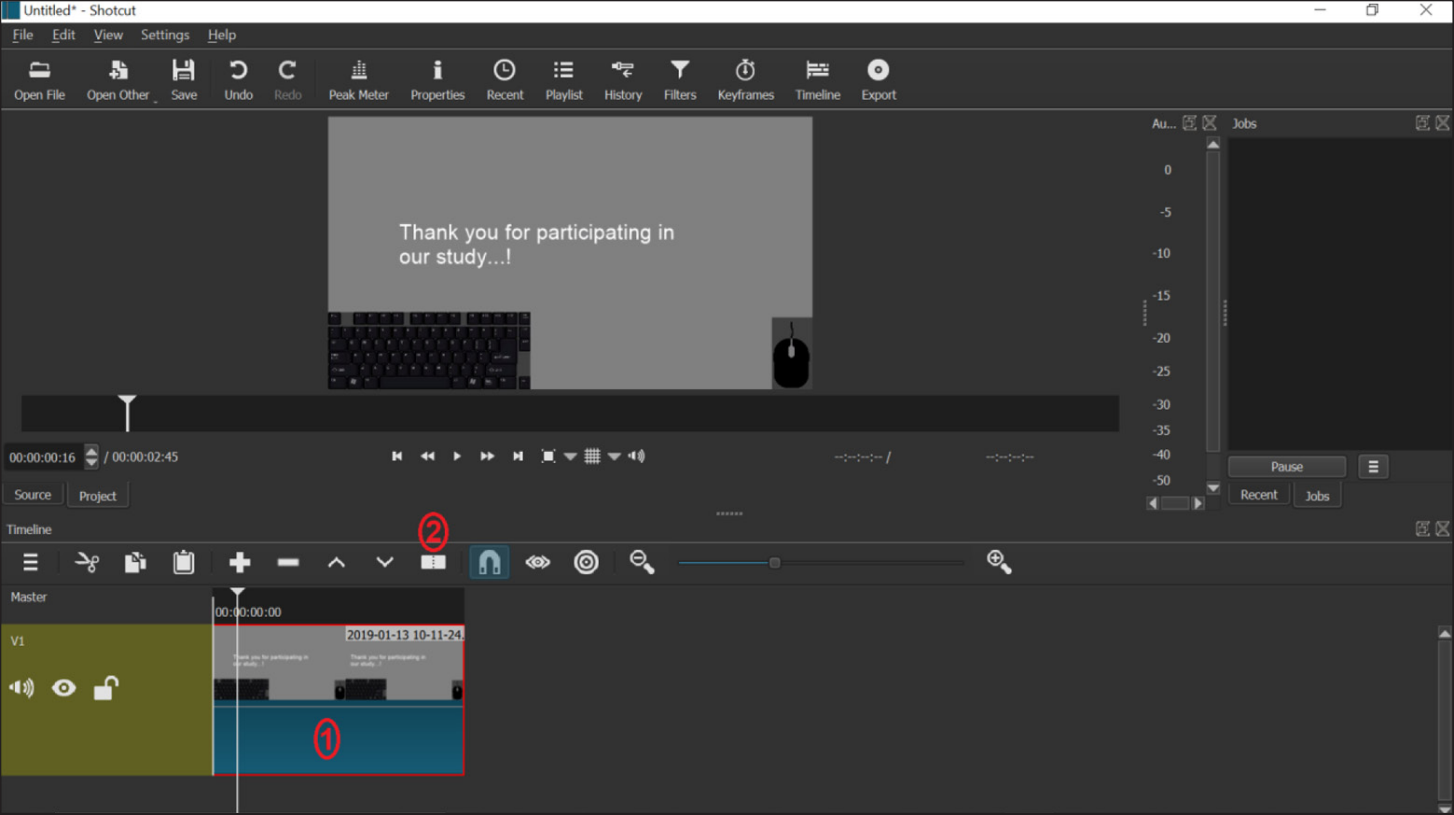
Screenshot of the ‘Shotcut’ software. **1)** At the bottom of the screen, the timeline of the video will be displayed and can now be edited. **2)** By clicking on the ‘split’ button, the video can be split into two parts and the irrelevant part can be removed.

When playing the video, a bar will move across this timeline representing the point in time of the video you are currently watching. One can move this bar manually with the mouse or by using the arrow keys on the keyboard to select specific points of the video. This way, one can move to the point in time where the actual experimental procedure starts. After selecting this point, one should click on the “split” button above the video timeline (see Figure 2.2). The video will now be split into two parts, 1) the irrelevant beginning and 2) the experiment (and the irrelevant ending that also needs to be removed later). Now, one can simply select the irrelevant part, right-click it and select the option “Remove”. The irrelevant part will be deleted and the video will now start with the beginning of the recorded experiment. The irrelevant ending can be removed in an analogous manner. After this editing process, the only thing left to do is to export the video (per default, it will be exported as mp4).

## Uploading

The last step of the screen recording process should be to upload the video and thus making it accessible for other scientists. Of course, each researcher is free to upload their video to any possible website/hosting option of their liking (e.g., personal website, youtube etc.). However, there might be some things that need to be taken into consideration when uploading it to a given platform. For example, when uploading to a personal website, the website might be removed when a researcher leaves academia and the video might not be accessible anymore. We would therefore suggest to use the Open Science Framework (OSF, https://osf.io) as it is a website designed for archiving research material, the file can easily be uploaded just like any other file, and it supports the suggested mp4 format so the video can be played within the web browser (for an example of an experimental recording uploaded to OSF, see https://osf.io/eyfxs/).

## General Discussion

As discussed in the introduction, replications are necessary to gain trust in an effect and the phenomenon it describes. We furthermore argued that direct replications are more informative than conceptual replications in an initial stage of replication as null effects can be attributed to a change of methods in conceptual replications. Problematically, not all details of the experimental procedure might be included in the published manuscript as researchers are potentially not aware which details might moderate the effect. The lack of information might not only be problematic for replication attempts, but might also hinder peer reviewers and others interested in the procedure to fully understand and criticize the used method. Even though researchers started uploading their experimental material to public repositories, we argue that these materials should be accompanied by a screen recording of an example procedure. Video recordings are easy to view by other researchers and do not depend on research software. Importantly, as we have shown above, screen recordings of experimental procedures are easy to record and upload and therefore constitute a nice addition to the written report and the research material.

As any documentation method, screen recordings have some shortcomings that should be mentioned. First, if presentation time is an essential part of the procedure (e.g., with brief and sub-optimal stimulus presentation), it should be noted that presentation times depend on both the refresh rate of the video recording and the refresh rate of the monitor the video is viewed on. Therefore relatively large differences in stimulus presentation times between the actual experimental procedure and the video can be expected. We advise researchers who provide videos with sensitive stimulus timings to warn viewers about this issue in the video or when providing the link. Second, problems with the stimulus randomization or counterbalancing cannot be detected when viewing one example video. Based on these two shortcomings, screen recordings should therefore only be viewed as an additional tool to document the data collection procedure. Refresh rates and computer settings still need to be reported in the manuscript and materials such as the experimental script should be provided. Third, colors might differ between different screens and a video on its own might not be informative. If the colors of stimuli on the screen are of high importance the material necessarily needs to be provided. Fourth, videos in different languages might make it difficult to understand the procedure. However, merely supplying the experimental material suffers from the same problems. In all instances, the video can nevertheless give some ideas on the procedure, timing, and scales used, which should be accompanied by translations, additional material, and experimental scripts. Of course, it should be mentioned that screen captures are limited to computer assisted data collection procedures.

To sum up, we have outlined why experimental procedures should be described as closely as possible and we postulate that screen recordings of an example procedure offer a simple and detailed opportunity for such a documentation. We additionally provided a brief tutorial on an open source screen recording software and hope to have motivated others to use this tool to document their experimental procedures. We would like to engage researchers in a discussion on possibilities to better document their experimental procedures and see this tutorial as a first starting point. We are certain that the process can be improved upon and look forward to this discussion.

## Footnotes

We use the word ‘experiment’ for any computer assisted data collection procedure, regardless of its research design.
